# Impact of intensified prevention measures on the rate of hospital-acquired bloodstream infections among mechanically ventilated COVID-19 patients

**DOI:** 10.1017/ash.2023.505

**Published:** 2023-12-14

**Authors:** Shimrit Lampl, Yael Cohen, Yasmin Maor, Debby Ben-David

**Affiliations:** 1 Wolfson Medical Center, Holon, Israel; 2 Faculty of Medicine, Tel Aviv University, Tel Aviv, Israel

## Abstract

**Background:**

The COVID-19 pandemic was associated with increased rates of hospital-acquired infections. During the early months of the pandemic, we observed high rates of hospital-acquired bloodstream infections (HA-BSIs) among COVID-19 patients, prompting the implementation of intensified prevention measures.

**Objectives:**

To assess the prevalence of HA-BSI among mechanically ventilated COVID-19 patients, identify risk factors, and evaluate the effect of prevention measures.

**Methods:**

We conducted a retrospective matched case-control study in adult medical step-up units between March 1, 2020, and March 31, 2021. We matched mechanically ventilated COVID-19 patients with ventilated non-COVID-19 patients based on age group and length of stay before ventilation. In response to the high rates of HA-BSI among COVID-19 patients, a comprehensive infection control intervention was implemented.

**Results:**

A total of 136 COVID-19 patients were matched with 136 non-COVID-19 patients. No significant differences were observed in pre-hospitalization characteristics. The central venous catheter utilization ratio was higher in COVID-19 patients (83.6%) versus 35.6% in the control group (*p* < 0.001). During pre-intervention, 35.2% (32/91) of COVID-19 patients developed HA-BSI, compared to 17.8% (13/73) in the control group (*p* < 0.001). Following the intervention, no significant difference was observed between the groups (17.8% (8/45) versus 15.9% (10 /63), *p* = 0.79). In a multivariate analysis, HA-BSI was associated with low body mass index (OR 0.9 (95% CI 0.9–1.0), *p* = 0.015)) and presence of temporary dialysis catheter (OR 2.7 (95% CI 1.0–7.3), *p* = 0.05)).

**Conclusions:**

Mechanically ventilated COVID-19 patients were at higher risk for developing HA-BSI compared to non-COVID-19 patients. Intensified prevention measures were associated with decreased rates of HA-BSI.

## Introduction

The emergence of COVID-19 had a profound impact on healthcare systems worldwide.^1^ The increased demand for healthcare systems, changes in patient management, increased use of personal protective equipment, and increased utilization of antibiotics are all factors that can impact the risk of healthcare-associated infections (HAIs).^2^ During the first year of the pandemic, there were multiple waves of illness that contributed to a significant rise in hospitalizations of patients experiencing critical respiratory failure.^3^ Simultaneously, a notable decline in the proportion of hospitalizations for patients with mild or elective conditions was observed.^4^ Throughout this period, hospitals faced a shortage of healthcare personnel due to both illness and extended periods of isolation of staff who were exposed to COVID-19.^5^ During the initial months of the pandemic, infection prevention teams faced numerous challenges in their efforts to effectively respond to the outbreak. These challenges encompassed various tasks, including the development of guidelines, staff training, and conducting epidemiological investigations to assess exposures within hospitals. Consequently, routine infection prevention activities, such as monitoring HAIs and conducting ward audits, experienced significant reductions due to the overwhelming demands placed on the teams.^2,6^ As anticipated, during the initial months of the COVID-19 pandemic, numerous healthcare facilities worldwide witnessed a notable increase in the overall rate of HAIs.^2,7^ This increase was particularly pronounced in hospitals with a higher prevalence of COVID-19 cases.^8^

Previous studies have reported high rates of HAI among critically ill patients with COVID-19.^9–11^ In a multicenter study conducted in Italy, almost half of patients in intensive care units (ICUs) experienced an infectious episode during their ICU stay.^9^ The most common HAIs were ventilator-associated infections and hospital-acquired bloodstream infections (HA-BSIs). The increased risk of HA-BSI among COVID-19 patients may be attributed to the increased severity of the disease, prolonged length of stay, high prevalence of central venous catheter (CVC) use, and high-dose steroid therapy.^12^ Limited data are available regarding the impact of targeted infection control interventions on the incidence of HA-BSI among critically ill COVID-19 patients. Hence, the objectives of our study were to evaluate the prevalence of HA-BSIs among mechanically ventilated COVID-19 patients compared with non-COVID-19 patients, identify risk factors for the first episode of BSI, and evaluate the effect of the implementation of prevention measures.

## Methods

### Study setting

Wolfson Medical Center is a 670-bed secondary-care teaching hospital located in central Israel. The hospital has six medical wards, each of which includes a step-up unit (SUU) with five critically ill patients hospitalized in a multi-bed open room. During the COVID-19 pandemic, one of the SUUs was dedicated to the care of critical COVID-19 patients.

### Study design

This was a retrospective matched case-control study conducted between March 1, 2020, and March 31, 2021. The study included all patients who required mechanical ventilation for ≥48 hours due to SARS-CoV-2 infection, confirmed by reverse transcription polymerase chain reaction on nasopharyngeal swabs. The control group consisted of non-COVID-19 patients who required mechanical ventilation. Each case was matched to one control based on age (±5 years) and duration of stay before ventilation. The inclusion criteria were as follows: (a) adults aged 18 years or older, and (b) mechanical ventilation for ≥48 hours. We assessed risk factors for HA-BSI among mechanically ventilated patients for ≥48 h prior to onset of HA-BSI, considering only the first episode of BSI. We excluded patients with community-onset BSIs, and all patients with mechanical ventilation <48 hours. Hospital-acquired bloodstream infections were classified as central line-associated bloodstream infection (CLABSI), secondary BSI, or primary non-CLABSI (laboratory-confirmed BSI that is not secondary to an infection at another body site) using the National Healthcare Safety Network definitions.^13^ For all study patients, the following characteristics were collected: age, sex, medical history, body mass index (BMI), and previous hospitalization during the past month. Events during hospitalization included CVC use, site of CVC, and steroid treatment.

### Description of the intervention

In response to the high rates of HA-BSI among COVID-19 patients, from September 2020 onward, we initiated a continuous, multimodal intervention strategy. The key components of the program included:

**Healthcare provider training**: Physicians and nursing staff underwent training on aseptic techniques, use of ultrasound during CVC insertion, and the importance of avoiding femoral insertion sites and proper maintenance practices. The program incorporated simulation-based sessions for both CVC insertion and maintenance procedures. Given the high turnover of staff, multiple training sessions were conducted.

**Maintenance protocols:** Implementation of daily assessments to determine the necessity of CVCs and evaluations of catheter dressing integrity. Additionally, a daily regimen of chlorhexidine bathing was introduced, along with the routine use of chlorhexidine-impregnated dressings and alcohol port protectors.

**Auditing:** To ensure high compliance with these preventive measures, infection control preventionists conducted bi-weekly audits, primarily focused on catheter maintenance practices.

The results are presented as frequencies (%) for categorical variables or median (25th, 75th quartiles) for continuous variables. Risk factors for HA-BSI were compared between the groups during pre-intervention period (March 2020–October 2020), intervention period (November 2020–March 2021), and throughout the entire study period. Categorical variables were compared using the χ^2^ test or Fisher’s exact text. Continuous variables that were normally distributed were compared using independent-sample t-tests. Odds ratios (ORs) and associated 95% confidence intervals (CIs) were calculated. Variables with an unadjusted *P* value less than 0.1 were entered into the multivariate binary logistic regression analysis to obtain adjusted P values. Risk factors with a *P* value of 0.05 and OR greater than 1 were considered significant risk factors. All data were analyzed using SAS version 9.4. The study was approved by the jurisdictional board review of the institution.

## Results

During the first 12 months of the pandemic, 136 COVID-19 patients requiring ventilation for ≥48 hours were hospitalized in the internal medicine-coronavirus SUU. During the same period, 450 non-COVID-19 patients requiring ventilation for ≥48 hours were hospitalized in the medical SUU, of whom 136 patients were matched based on age and ventilation duration.

Table 1 displays the demographic and clinical characteristics of patients with and without COVID-19 prior to hospitalization. The median age in both groups was 79 (IQR 69–86), and 53.7% were male. COVID-19 patients exhibited a lower prevalence of chronic lung disease compared to non-COVID-19 patients (15.4% vs. 25.2%, respectively, *p* = 0.046). There were no significant differences in the prevalence of other comorbidities, mean BMI, functional status before admission, or Charlson Comorbidity Index.

**Table 1.** Demographic and clinical characteristics prior to hospitalization—comparison between COVID-19 and non-COVID patients

Variable	COVID-19 patients *n* = 136	Non COVID-19 patients *n* = 136	*P*-Value
Male	73 (53.7)	63 (46.3)	0.225
Age (years)	79 (69–86)	79.5 (69–87)	0.857
Transfer from long-term care facility	46 (33.8)	32 (23.5)	0.061
Charlson Comorbidity Index	2 (0–3)	2 (0–4)	0.273
Prior hospitalization within 1 month	48 (35.6)	45 (33.1)	0.669
Dementia	40 (29.4)	40 (29.4)	1.000
Dependent functional status	53 (39.3)	62 (45.6)	0.444
BMI	27 (25–30)	26 (23–31)	0.169
CHF	44 (32.4)	53 (39.3)	0.236
COPD	21 (15.4)	34 (25.2)	0.046

Note. Data is presented as median (25%, 75% quartiles) or *n* (%). BMI, body mass index; CHF, congestive heart failure; COPD, chronic obstructive pulmonary disease.

Table 2 describes the characteristics of patients during hospitalization. The length of stay before ventilation among COVID-19 patients was 5.9 ± 6.1 days, which did not significantly differ from non-COVID-19 patients (*p* = 0.26). The utilization of CVC was more common among COVID-19 patients (83.6%) compared to non-COVID-19 patients (35.6%, *p* < 0.001). A higher prevalence of steroid treatment was observed among COVID-19 patients (52.9% vs. 6.6%, respectively, *p* < 0.001). The most frequent site of CVC insertion was the femoral site, used in 52.5% (62/112) of COVID-19 patients and 53.8% (26/48) of non-COVID-19 patients (*p* = 0.144). Before the intervention, the CVC utilization rate among COVID-19 patients was 84.6% (77/91) compared to 77.8% (35/45) after the intervention (*p* = 0.311).

**Table 2.** Characteristics during hospitalization—comparison between COVID-19 and non-COVID mechanically ventilated patients

Variable		COVID-19 patients *n* = 136	Non COVID-19 patients *n* = 136	*P*-Value
LOS before mechanical ventilation, median (days)		4 (2–8)	4 (2–7)	0.266
Steroid treatment		72 (52.9)	9 (6.6)	<.001
Presence of CVC		112 (83.6)	48 (35.6)	<.001
Site of CVC	Femoral	62 (52.5)	26 (57.8)	0.144
	Jugular	1 (43.2)	14 (31.1)	
	Subclavian	5 (4.2)	5 (11.1)	
Temporary dialysis catheter		10 (7.4)	12 (9.2)	0.591
Presence of indwelling urinary catheter		28 (96.6)	21 (87.5)	0.318

Note. Data is presented as median (25%, 75% quartiles) or *n* (%). CVC, central venous catheter; LOS, length of stay.

Throughout the entire study period, a total of 29.4% (40/136) of COVID-19 patients developed HA-BSI, compared to 16.9% (23/136) of patients in the control group (*p* = 0.015). The median time from ventilation to the development of HA-BSI was 6 days (IQR 4–11) in the COVID-19 group compared with 10 days (IQR 6–14) in the control group (*p* = 0.182). The causes of HA-BSI are detailed in Table 3, with 55.9% attributed to pneumonia (40/22) and 30% to the presence of CVC (12/40). Among the control group, primary non-CLABSI BSI was the most common type of HA-BSI, accounting for 60.9% (14/23).

**Table 3.** HA-BSI rate, length of hospital stay, and mortality—comparison between COVID-19 patients and non-COVID-19 mechanically ventilated patients

Parameter		COVID-19 patients *n* = 136	Non COVID-19 patients *n* = 136	*P*-Value
HA-BSI (%)		40 (29.4)	23 (16.9)	0.015
Source of BSI *n* (%)	Pneumonia	22 (55.9)	4 (17.4)	0.016
	CLABSI	12 (30.0)	2 (8.6)	
	Primary	2 (5.0)	14 (60.9)	
	Other	4 (10.0)	3 (13.0)	
Duration of ventilation before onset of BSI (days)		6 (4–11)	10 (6–14)	0.182
LOS, (days)		14 (8–25)	17 (9–27)	0.272
Mortality		110 (80.9)	82 (60.3)	<.001

Note. Data is presented as median (25%, 75% quartiles) or *n* (%). HA-BSI, hospital-acquired bloodstream infection; CLABSI, central line-associated bloodstream infection; LOS, length of stay.

Before the intervention, 35.2% (32/91) of COVID-19 patients developed HA-BSI compared to 17.8% (13/73) of non-COVID-19 patients (*p* = 0.015). Following the intervention, no significant difference was observed between the two groups (17.8% (8/45) for COVID-19 patients and 15.9% (10/63) for non-COVID-19 patients (*p* = 0.79).

Table 4 presents the results of the univariate analysis of risk factors for the development of HA-BSI. In the multivariate analysis conducted for the entire study period, low BMI (*p* = 0.015; OR 0.9 (95% CI 0.9–1.0)) and the presence of a temporary dialysis catheter (*p* = 0.05; OR 2.7 (95% CI 1.0–7.3)) were found to be associated with the development of primary HA-BSI (Table 5). Prior to the intervention, COVID-19 was associated with an increased risk of developing HA-BSI (*p* = 0.015; OR 2.5 (95% CI 1.2–5.2)). However, following the intervention, the presence of a CVC (*p* = 0.024; OR 4.6 (95% CI 1.2–17.1)) and low BMI (*p* = 0.001; OR 0.7 (95% CI 0.6–0.9)) were identified as independent risk factors for the development of HA-BSI.

**Table 4.** Univariate analysis of predictors of HA-BSI

		Before intervention		After intervention		Both periods	
Parameter		OR (95% CI)	*P*-Value	OR (95% CI)	*P*-Value	OR (95% CI)	*P*-Value
Age		1.0 (1.0–1.0)	0.211	1.0 (0.9–1.0)	0.555	1.0 (1.0–1.0)	0.167
Gender Male		1.3 (0.6–2.5)	0.516	3.0 (1.0–8.7)	0.044	1.7 (1.0–3.1)	0.063
Charlson Comorbidity Index		1.1 (0.9–1.3)	0.453	0.9 (0.7–1.2)	0.593	1.0 (0.9–1.2)	0.706
BMI		1.0 (0.9–1.0)	0.346	0.8 (0.7–0.9)	0.002	0.9 (0.9–1.0)	0.015
COVID-19		2.5 (1.2–5.2)	0.015	1.1 (0.4–3.2)	0.794	2.0 (1.1–3.7)	0.016
Presence of CVC		1.8 (0.9–3.9)	0.112	2.7 (0.9–8.1)	0.085	2.2 (1.2–4.1)	0.014
Temporary dialysis catheter		2.0 (0.7–5.6)	0.186	3.4 (0.5–22.1)	0.197	2.4 (1.0–6.0)	0.052
Insertion site of CVC	Jugular vs femoral	2.2 (1.0–5.3)	0.175	1.3 (0.4–4.4)	0.935	1.9 (0.9–3.8)	0.177
	Subclavian vs femoral	1.2 (0.2–6.9)		0.0 (0.0–0.0)		0.8 (0.2–4.1)	

Note. BMI, body mass index; CVC, central venous catheter; OR, odds ratio; CI, confidence interval.

**Table 5.** Multivariate logistic regression analysis of risk factors for hospital-acquired bloodstream infection among mechanically ventilated patients

A. Entire study period		
Parameter	OR (95% CI)	*P*-Value
BMI	0.9 (0.9–1.0)	0.008
Presence of temporary dialysis line	2.7 (1.0–7.3)	0.050
Pre-intervention period	1.6 (0.9–3.1)	0.130
Presence of CVC	1.7 (0.8–3.7)	0.160
COVID-19	1.6 (0.8–3.3)	0.177
Male gender	1.4 (0.7–2.6)	0.300

B. Pre-intervention period		
Parameter	OR (95% LCL–95% UCL)	*P*-Value
COVID-19	2.5 (1.2–5.2)	0.015

C. Intervention period		
Parameter	OR (95% LCL–95% UCL)	*P*-Value
BMI	0.7 (0.6–0.9)	0.001
Presence of CVC	4.6 (1.2–17.1)	0.024
Male	2.4 (0.7–8.3)	0.159

Note. BMI, body mass index; CVC, central venous catheter; OR, odds ratio; CI, confidence interval; LCL, low confidence limits; UCL, upper confidence limits.

All-cause in-hospital mortality was significantly higher in COVID-19 patients than in the control group (80.9% vs. 60.3%, *p* < 0.001). Among COVID-19 patients who developed HA-BSI, mortality was 82.5% (33/40) compared to 80.2% (77/96) among patients without HA-BSI (*p* = 0.15). Similarly, in the control group, there was no statistically significant difference in mortality between patients with HA-BSIs (65.2%, 15/23) and those without (58.8%, 67/114) (*p* = 0.46).

## Discussion

Consistent with prior studies, we observed higher rates of HA-BSI among COVID-19 patients. Additional significant risk factors for the development of HA-BSI were low BMI and the presence of a temporary dialysis catheter. Following the implementation of preventive measures targeting central venous catheter care, a reduction in the rate of HA-BSIs was observed among COVID-19 patients.

Our study revealed a notably high HA-BSI prevalence rate of 30% among patients with COVID-19. In a systematic review assessing the risk of developing HA-BSIs among COVID-19 patients, a prevalence rate of 7.3% was reported.^12^ However, most of these studies included populations of varying degrees of severity, including non-ventilated patients. Among patients who required hospitalization in the ICU, a prevalence of 30% was reported.^12^ Mantzarlis et al. observed a high incidence of HA-BSI exceeding 50% among COVID-19 mechanically ventilated patients.^14^ These findings underscore the heightened risk of HA-BSI in critically ill COVID-19 patients and emphasize the need for targeted preventive strategies in such high-risk patients. Notably, most HA-BSIs among the control group were defined as primary non-CLABSI. These infections could likely be attributable to short-term peripheral vascular catheters.^15^ This underscores the need to carefully monitor and manage short-term PVCs, which might sometimes be overlooked in favor of more central and long-term catheters.

Previous studies investigating the risk factors associated with the development of HA-BSIs among patients with COVID-19 have predominantly focused on individual factors, notably the requirement for invasive ventilation and administration of immunomodulating medications.^16,17^ Nonetheless, several studies conducted prior to the COVID-19 era have demonstrated the paramount significance of adherence to intravenous catheter care bundles in decreasing the risk of HA-BSI. Even in the presence of multiple risk factors, a systematic and rigorous approach to infection prevention, including the implementation of care bundles, can lead to a marked reduction in infection rates.^18^ Achieving high compliance rates, ideally above 90%, with prevention practices is crucial in this regard.^19^ The influx of COVID-19 patients has strained healthcare systems, potentially leading to lapses in infection control practices. Increased patient volume and the need for rapid care may have compromised proper catheter insertion and maintenance practices. We implemented a comprehensive intervention to address the high rates of HA-BSIs among COVID-19 patients. After our intervention, which included evidence-based practices for catheter insertion and maintenance, a notable reduction in HA-BSI rates was observed. These findings provide evidence that the elevated incidence of BSI among COVID-19 patients is primarily attributed to lapses in infection control practices rather than solely to individual risk factors or patient complexity.

The current study examined the risk factors for the development of HA-BSI among mechanically ventilated patients. Low BMI and the presence of a temporary dialysis catheter were found to be independent risk factors. In studies conducted on non-COVID-19 patients, the presence of a dialysis catheter was previously identified as a significant risk factor associated with the development of HA-BSIs among ICU patients.^20^ The association between BMI and the development of HA-BSIs remains controversial, with conflicting evidence in the literature. A recent meta-analysis focusing on ICU patients revealed that high BMI was independently associated with an increased risk of CLABSI.^21^ In contrast, in elderly patients over the age of 75, low BMI has been identified as a risk factor for the development of infections and mortality.^22,23^ Research conducted among patients with COVID-19 demonstrated that individuals with a lower BMI exhibited an elevated susceptibility to developing secondary infections.^24^

Consistent with a previous report, our data underscore a marked preference for the femoral site for CVC insertions among COVID-19 patients.^25^ This choice was largely driven by the urgency for immediate catheterization and efforts to reduce healthcare provider exposure. The femoral site’s distance from respiratory droplets potentially reduces the risk of exposure. Additionally, during the peak of the pandemic, numerous non-ICU staff, who might possess less experience with CVC placement, were involved in care, potentially influencing site selection. These challenges underscore the importance of establishing specialized intravenous teams for future public health crises.

Among patients with COVID-19, pneumonia was identified as the predominant source of BSIs. The diagnosis of pneumonia was established based on the criteria outlined by the NHSN, which define a pulmonary source when there is evidence of pulmonary infiltrates accompanied by respiratory and infectious signs.^26^ However, given that most patients were admitted to the hospital with pulmonary infiltrates, the development of BSIs among COVID-19 patients is frequently attributed to pneumonia. Nevertheless, the observed decrease in BSI rates following an intervention targeting the improvement of vascular catheter treatment procedures suggests that some cases previously categorized as pneumonia-related may in fact be associated with the presence of a venous catheter.

In the present study, the patient sample had a significantly higher age distribution, with a median age of 79 years, in comparison to previous studies that primarily encompassed patients below the age of 65.^27,28^ This observed increase in age distribution may serve as a contributing factor to the elevated mortality rates observed. Mortality rates among patients with HA-BSI did not show a significant increase when compared to patients who did not develop HA-BSI. The implications of BSIs on mortality in patients with COVID-19 have yielded conflicting reports. While some studies have identified HA-BSI as a risk factor for mortality compared to those without BSI,^29^ other studies have reported no significant increase.^14,27^ It is noteworthy that in critically ill COVID-19 patients, the complex pulmonary course and multisystem failure contribute to exceptionally high mortality rates, irrespective of the presence of BSI.

Our study has certain limitations. First, the study was conducted at a single center, which may limit the generalizability of the findings. While patients were matched based on age and ventilation duration, other relevant confounding factors that could influence outcomes may not have been fully accounted for. In addition, the study covers the first 12 months of the pandemic, and changes in treatment protocols or infection control practices during this period may affect the interpretation of the results. The follow-up period after the intervention in our study was relatively short, which limited our ability to assess the long-term effects of the intervention. Notably, we lacked data on catheter days in the units involved, which prevents evaluating the intervention’s effect on device use ratio.

In conclusion, in this study, we showed that mechanically ventilated COVID-19 patients were at high risk of developing HA-BSI compared to non-COVID-19 patients. Implementing preventive measures may lead to a decrease in the risk of HA-BSI. Within the framework of preparedness programs for future public health crises, it is essential to incorporate evidence-based strategies and interventions aimed at minimizing the risk of HAI within healthcare settings.
